# Treatment of Erysipelas

**Published:** 1848-11

**Authors:** 


### ECLECTIC DEPARTMENT,

### AND SPIRIT OF THE MEDICAL PERIODICAL PRESS.

---

To the Editor of the Boston Medical and Surgical Journal.

*Treatment of Erysipelas.*

Dear Sir,—I send you, for insertion in your Journal, that portion of an address delivered before the Worcester County Medical Society, which gives an account of my treatment of a few cases of erysipelas.

In August, 1846, I was called to E. C., a female aged 17, of sanguine temperament, florid complexion, auburn hair, had always been in perfect health. She was attacked with chills and fever, caused, as she supposed, by taking cold. She complained of severe cephalalgia. The mind sluggish, and a tendency to forget a question before she would be ready to answer. The tongue was dry and brown, with extreme heat of skin. I attributed much of the disturbance of the system to the fact that it was the first day of her menstrual period, and the discharge was stopped by her taking cold. I ordered an emetico-cathartic, composed of cal., pulv. sang. and ipecac., and followed it with diaphoretics and alteratives. There was a mitigation of all the fever symptoms, with the exception of the pain in the head, which continued with but little abatement. On the fourth day the erythematic inflammation made its appearance between the eyes, and extended over the nose and upon the forehead. It was first discovered soon after my visit in the morning, and I did not see it until evening.

I then applied nit. arg. around the inflamed surface, and slightly upon it; kept the face cool with spirits Mindereri. In the morning the inflammation had passed over the parts to which the caustic had been applied. I now painted it over with the alcoholic solution of iodine, but this did not check the progress of the disease; it advanced at a uniform rate. In the afternoon I called in my friend Dr. John Green, of Worcester. He considered the case a grave one, and advised a continuance of the iodine, and a free use of the saline cath., sufficient to produce several dejections during the twenty-four hours. This advice was followed strictly. Through the next day, the inflammation advanced as rapidly as before, the treatment apparently not equal in power to the disease.

From the circumstance that I had seen this form of erysipelas mostly among intemperate persons, in hospital practice, at Bellevue, N. Y., whose constitutions were impaired by their habits of indulgence and exposure, rendering them not only trying cases to the physician, but frequently unfortunate, I was anticipating anything but a favorable issue. I mention this as a reason why I had not much confidence in any plan of treatment, and was prepared to adopt any course which might seem to possess power sufficient to control the disease.

In reviewing the medication recommended, most of which I had used and seen used, I found that a majority of it had a tendency to diminish the temperature of the parts to which it was applied; and that I

had experienced the best results from that form which effected this object the most perfectly. Reasoning from these facts, I came to the conclusion that I would carry the refrigerating process to that extent, that it would control the inflammation, or give rise to some unpleasant symptoms that contra-indicated its continuance. In order that a thorough trial might be made of checking the inflammation by reducing the heat of the parts below their natural standard, I added salt to the ice, which is the mixture by which cream is frozen in the manufacture of ice-creams. This was put in a vessel and covered with a blanket. I then had a change of folded cloths, so that while one set was upon the patient, the other was upon the ice. The first application allayed the pain, and this relief continued until the parts began to grow warm again, which at first was not more than three or four minutes. It being necessary to apply the cloths in strips, it was a continual process of change. When the inflamed part was kept so cold as to feel almost like ice to the hand of the nurse, the patient would sleep quietly, and was free from pain; but if the attendant neglected to change the cloths in a given time, the pain would wake the patient.

From the commencement of this treatment, the inflammation not only ceased to progress, but began to subside, the general symptoms improving in the same ratio. After a few hours the cloths required changing less often; and as the disease yielded, she complained that the application made her chilly. The sensations of the patient (so far as evidence can be derived from four cases*), are a safe guide upon this point. This case went rapidly on to perfect restoration without any untoward circumstance to mar the happy result, or tend to create distrust of the truth of my deductions.

February 28th, 1847, was called to a young man, aged 28, very fleshy, of temperate habits, and good general health. Four days before, he was taken unwell, complained of headache, chills and general symptoms of fever. The pain in his head increased, until he was unable to work. This was on Saturday, the third day. His wife gave him a sweat, as she said; he perspired freely, and the head was relieved, but in the morning she discovered redness and swelling at the inner canthus of both eyes, and across the nose. It was upon the evening of this, the fourth day, that I was called to see him, for the first time, and found the inflammation had extended over both eyelids. I administered the same general remedies, and painted the inflamed surface over with iodine, made cooling applications, circumscribed the diseased portion with nit. argenti, keeping it upon the healthy skin. This caused vesication, but did not prevent the progress of the disease.

I now had recourse to the freezing mixture, as described in the other case. The general symptoms being so bad, I desired counsel, and Dr. Green was sent for. On his arrival, he advised a continuance of the treatment, as it had been so successful in the other case. The inflamed surface was excessively hard; it had, as Dr. Green remarked, a woody feel This extreme refrigeration checked the spread of the inflammation, and the recovery was rapid. On the fourteenth day from the attack, after eating a

---

* One since, making five.

hearty dinner and taking considerable exercise, the cheek began to inflame, but it was controlled immediately by the application of ice.

May 20th, 1848, Saturday, was called to see a young miss, aged 11, in consultation. She had been under the care of Dr. Corlew from the Wednesday preceding, at which time the erythematic inflammation made its appearance, and first upon the nose between the eyes. The usual treatment had been pursued from Wednesday, until the time of my seeing her on Saturday about 1 o'clock, P. M. At this time the whole face, ears, and about two and a half inches of the scalp, were inflamed and much swollen, so that it was with great difficulty the eyelids could be separated sufficiently to exhibit the eyeball; yet it had not that indurated feel of the last case. The lower extremities were cold, the upper portion of the body very hot. The pulse varied from 130 to 140, and was quite feeble. The pain in her head was most intense, accompanied with raving delirium; she did not recognize her parents, or any one about her, but was constantly exclaiming that "they had split her head open," "they had cut her head off," "they had killed her," &c., indicating the suffering she was undergoing.

Just after I had prescribed for the patient, Dr. Green arrived; and after making an examination, he stated to the attending physician, "that he had seen three or four cases that I had treated successfully in this way, and he must say more so than he had anticipated, and he should advise its continuance. The face having blistered in several places, the salt could not be borne, and cloths cooled only by the ice were used. Before leaving, gave four or five grains Dover's powder. At 5 o'clock, saw her again. She was quiet, had slept some, was calm and rational; said she had some pain in her head, but before I was through with examining the extent of the inflammation the pain became so intense that she cried out from the suffering. This was relieved as soon as the cold cloths were applied again. The bowels were moved several times a day by the Rochelle salts; she took an infusion of Asclepias tuberosa, with fifteen or twenty drops of spts. nit. dulc. every three hours. After a few days the rad. serp. virg. was added to the infusion.

On the eighth day from my first visit, all symptoms of fever left her, and her recovery was rapid. A small abscess of a strumous character formed on the inferior maxillary bone, and another upon the forehead; the latter did not require opening.

In all these cases the rooms were kept cool, and there was a free circulation of air.

There are some conditions which appear so essential in order to accomplish the object sought by this plan of treatment, that I will mention them.

1st. The temperature of the inflamed parts, and for some distance around, should be kept much below the natural standard.

2nd. Care should be taken that no small portion is overlooked, so that the inflammation may be extending itself unperceived at that point.

3d. The diseased part should be kept *constantly* cold.

If this last precaution is not taken, and the parts become hot, the inflammation will extend rapidly, and it will require some hours to recover what has been lost.

This treatment can be considered no other than topical, and sustains no different relation to the general or constitutional medication than any other local remedies; and I can perceive no objection to its being adopted, let the internal remedies be what they may. I should recommend the application of the alcoholic solution of iodine to the surface, as it serves to prevent vesication, and probably is of advantage beyond this, in changing the condition of the parts beneath the scarf skin, or cuticle, by reason of its acrid and alterative power.

In the last case where the disease had extended behind the ears, under the chin, and upon the neck, I found it difficult to apply cloths so as to reach all the parts and keep them constantly cold. These difficulties suggested the idea of suspending the head in a net, or what might be obtained in almost any family, and would answer as good a purpose, the article termed tidies. This mode of suspending the head would allow the pillows to be moved, and admit a piece of oil-cloth to conduct off the water; I then would place over the head of the patient a vessel containing ice and salt, the water from which would be let upon the head through a faucet, in such quantity as was desirable. The parts desired to be kept wet should be first covered with cloths.

In this way I think all of the conditions I mentioned could be complied with, and a favorable result secured, without the fatigue, anxiety, and, more than all, the liability of failure by reason of the unfaithfulness, or want of judgment, of attendants.

The temperature of the parts could be regulated by the quantity of water permitted to flow, or by the ice and salt, or by both together.

MILLBURY, Sept. 1st, 1848. H. G. DAVIS.

[*Boston Med. and Surg. Journal.*

